# Determinants of vitamin D status in Kenyan calves

**DOI:** 10.1038/s41598-020-77209-5

**Published:** 2020-11-25

**Authors:** Rebecca Callaby, Emma Hurst, Ian Handel, Phil Toye, Barend M. de C. Bronsvoort, Richard J. Mellanby

**Affiliations:** 1 The Epidemiology, Economics and Risk Assessment (EERA) Group, The Roslin Institute and The Royal (Dick) School of Veterinary Studies, Easter Bush Veterinary Centre, The University of Edinburgh, Roslin, EH25 9RG Midlothian UK; 2 Centre for Tropical Livestock Genetics and Health (CTLGH), The Roslin Institute, University of Edinburgh, Easter Bush Campus, Roslin, EH25 9RG UK; 3 The Vitamin D Animal Laboratory (VitDAL), The Royal (Dick) School of Veterinary Studies and The Roslin Institute, Easter Bush Veterinary Centre, The University of Edinburgh, Roslin, EH25 9RG Midlothian UK; 4 International Livestock Research Institute and Centre for Tropical Livestock Genetics and Health, Nairobi, Kenya

**Keywords:** Ecology, Immunology, Zoology, Ecology, Diseases, Endocrinology

## Abstract

Vitamin D plays a critical role in calcium homeostasis and in the maintenance and development of skeletal health. Vitamin D status has increasingly been linked to non-skeletal health outcomes such as all-cause mortality, infectious diseases and reproductive outcomes in both humans and veterinary species. We have previously demonstrated a relationship between vitamin D status, assessed by the measurement of serum concentrations of the major vitamin D metabolite 25 hydroxyvitamin D (25(OH)D), and a wide range of non-skeletal health outcomes in companion and wild animals. The aims of this study were to define the host and environmental factors associated with vitamin D status in a cohort of 527 calves from Western Kenya which were part of the Infectious Disease of East African Livestock (IDEAL) cohort. A secondary aim was to explore the relationship between serum 25(OH)D concentrations measured in 7-day old calves and subsequent health outcomes over the following 12 months. A genome wide association study demonstrated that both dietary and endogenously produced vitamin D metabolites were under polygenic control in African calves. In addition, we found that neonatal vitamin D status was not predictive of the subsequent development of an infectious disease event or mortality over the 12 month follow up period.

## Introduction

Vitamin D plays a critical role in calcium homeostasis and in the maintenance and development of skeletal health. Vitamin D can be obtained from dietary sources or from production in the skin^1^. Cattle can obtain vitamin D from ingestion of vitamin D_2_ or D_3_^2^. Vitamin D_2_ is present in some plants following the conversion of ergosterol to vitamin D_2_ by ultraviolet radiation. Cattle are only likely to consume dietary sources of vitamin D_3_ if they have access to proprietary foodstuffs which are directly supplemented with vitamin D_3_ or contains vitamin D_3_ rich ingredients such as oily fishes. Vitamin D_3_ can be produced cutaneously following the isomerisation of 7-dehydrocholesterol by ultraviolet radiation^3^. If insufficient vitamin D is consumed or produced cutaneously, skeletal complications can develop in cattle as a sequelae of prolonged and severe vitamin D deficiency^2^.

Although the importance of vitamin D has been recognised for several decades in the development and maintenance of skeletal health^1,4^, the role of vitamin D in non-skeletal health outcomes has been extensively explored following the discovery that many cell types express the vitamin D receptor^5,6^. Vitamin D metabolites have subsequently been shown to have extensive immunomodulatory roles in experimental models and human studies^7–9^. Low vitamin D status has been linked to all-cause mortality^10–12^ and numerous other infectious^13–15^, allergic^16^ and autoimmune diseases^17,18^ in humans. Vitamin D supplementation trials have indicated potential beneficial roles in cancer outcomes^19^ and reduction in diabetes risk in vitamin D deficient individuals^20^. Similarly in companion animals, low vitamin D status has been linked to mortality in hospitalised cats^21^ and dogs ^22^ and in animals with specific diseases such as dogs with chronic enteropathies^23,24^. Low vitamin D status has also been associated with inflammation in dogs^25^ and cats^26^.

The relationship between vitamin D and non-skeletal health outcomes has been less studied in ruminants. We have previously reported a positive association between autumnal serum 25(OH)D concentrations and subsequent fecundity^27^ and birth weight^28^ the following spring in sheep. Vitamin D metabolites have been shown to modulate bovine immune cell phenotype *in-vitro*^29–32^ and *in-vivo*^33,34^. In addition, vitamin D status has been found to be significantly lower in cattle with clinical *Mycobacterium avium paratuberculosis* disease compared to cows in a subclinical stage and non-infected control cows further supporting a potential role of vitamin D in regulating non-skeletal health outcomes^35^.

**Figure 1:**
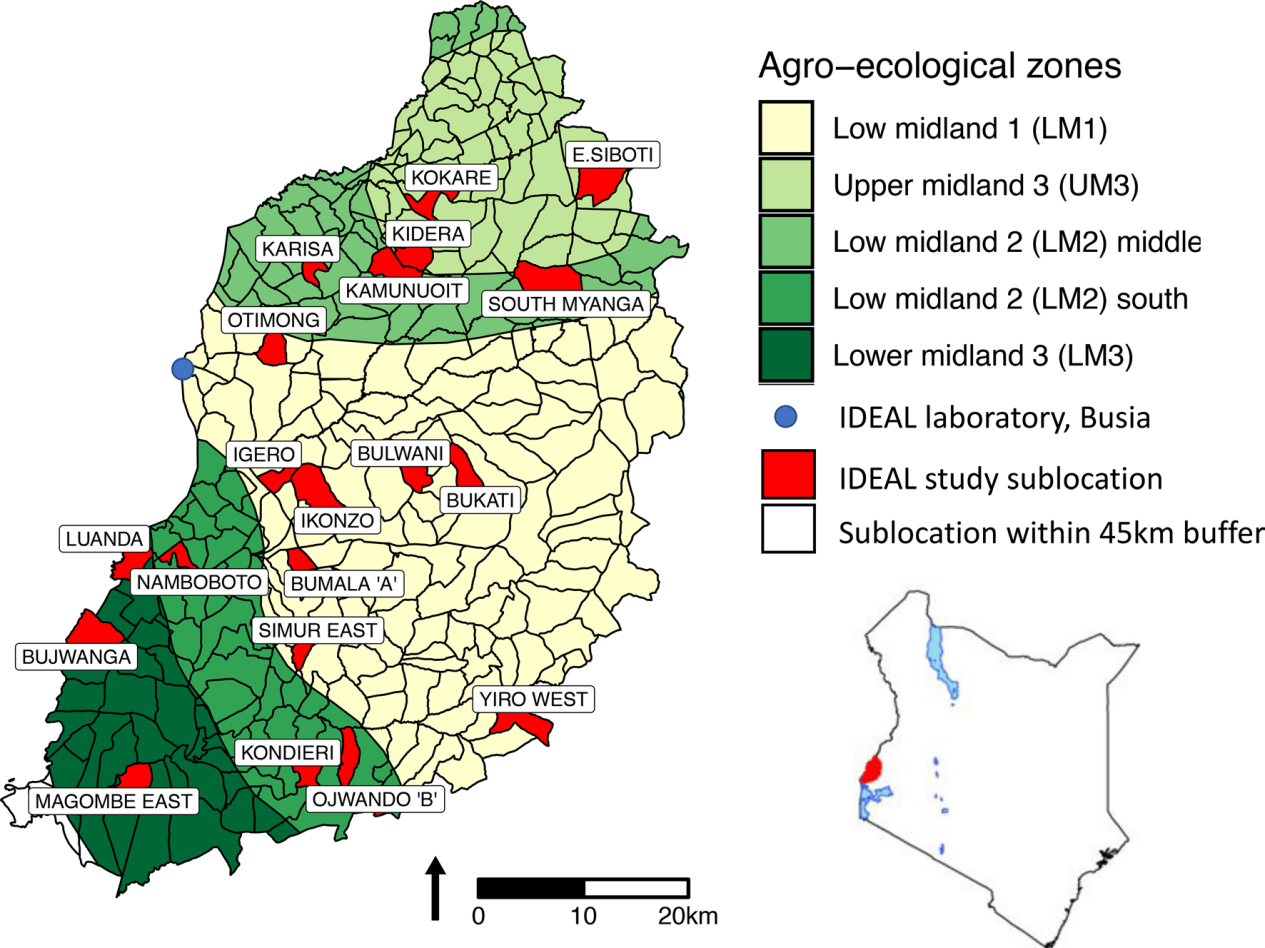
Map of western Kenya showing the study area, agro-ecological zones and sublocations. Selected sublocations are highlighted in red. Figure taken from Bronsvoort et al. (2013).

Despite the accumulating evidence that vitamin D may regulate non-skeletal health outcomes in cattle, little is understood about the genetic and environmental factors which are associated with vitamin D status. Furthermore, no studies have explored the relationship between neonatal vitamin D status and subsequent health outcomes in a population of large, healthy calves in a non-experimental, natural setting. We have addressed these research questions by measuring 25(OH)D concentrations in 527 calves from Western Kenya which were part of the precisely phenotyped Infectious Disease East African Livestock (IDEAL) cohort^36^, see Fig. 1 for a map of the study area. All calves in this cohort were examined by an animal health technician every 5 weeks and detailed information about infectious disease incidence was recorded. All calves were followed until 12 months of age or until death. This cohort provided us with an excellent opportunity in which to explore vitamin D regulation in cattle and its relationship with subsequent health outcomes.

## Results

### Cohort description

In total, serum total 25(OH)D, 25(OH)D_2_ and 25(OH)D_3_ concentrations were available from 527 calves which were included in this study. The cattle were all between 3–7 days old, East African Shorthorn Zebus and there were 278 males and 249 females. The cattle had a mixture of coat colours, 63 having a light coat, 88 having a dark coat, 174 having a brown coat, 194 having a mixed coat. Eight cattle did not have their coat colour recorded. In addition, 428 of the farmers rerolled in this study stated that they gave cattle in their herd nutritional supplements. Furthermore, the dams of the studied calves were mainly of normal body condition score (n = 366), or fat (n = 145), very few dams were lean (n = 16).

**Figure 2:**
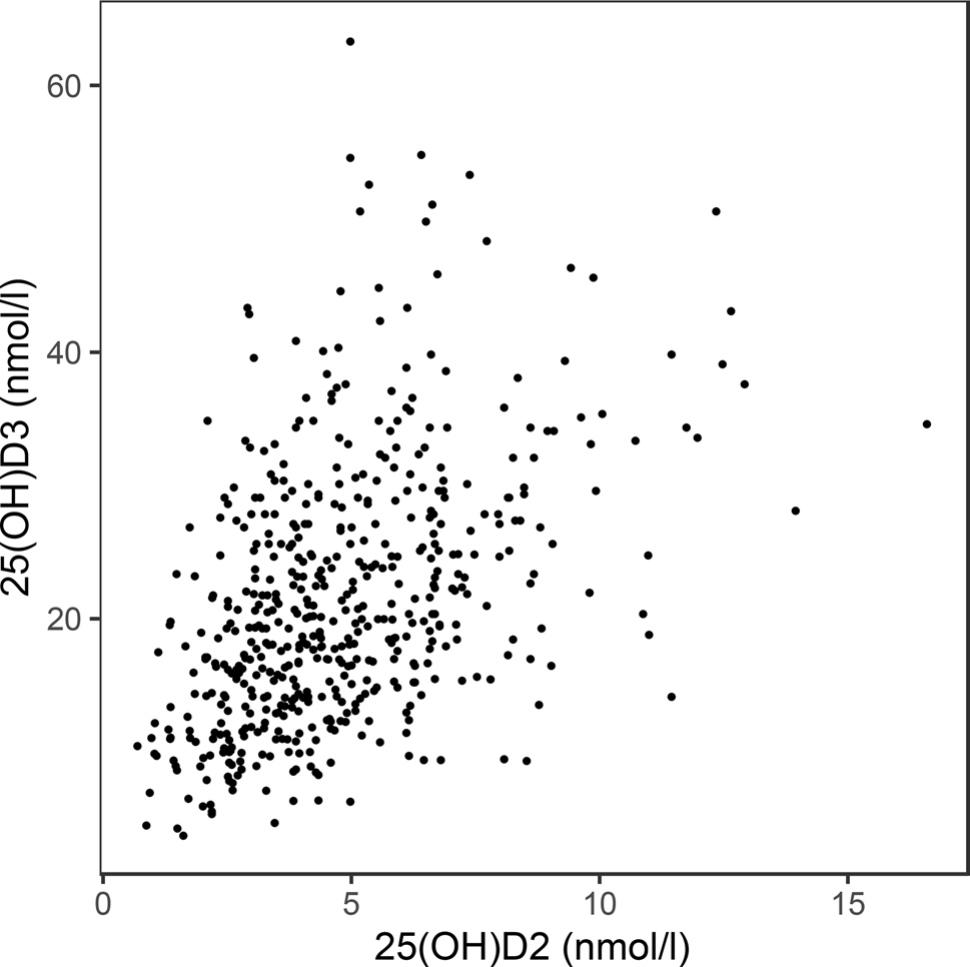
Scatterplot showing the relationship between 25(OH)D2 and 25(OH)D.

Serum 25(OH)D_2_ and 25(OH)D_3_ concentrations were positively correlated (Spearman rank correlation = 0.45, p < 0.001, Fig. 2). On average, 25(OH)D_3_ concentrations were 16.71 nmol/l (95% CI = 15.94–17.47 nmol/l) higher then 25(OH)D_2_ concentrations (Table 1).

**Table 1:** Descriptive summary of the mean serum 25(OH)D concentrations.

	Mean (nmol/l)	Standard error	Range (nmol/l)	Median (nmol/l)	Inter quartile range (nmol/l)
25(OH)D2	4.82	0.10	0.72—16.62	4.42	2.95
25(OH)D3	21.53	0.42	3.82—63.40	19.79	12.90
Total 25(OH)D	26.36	0.48	5.46—68.42	24.66	13.77

### Association with calf level factors

**Figure 3:**
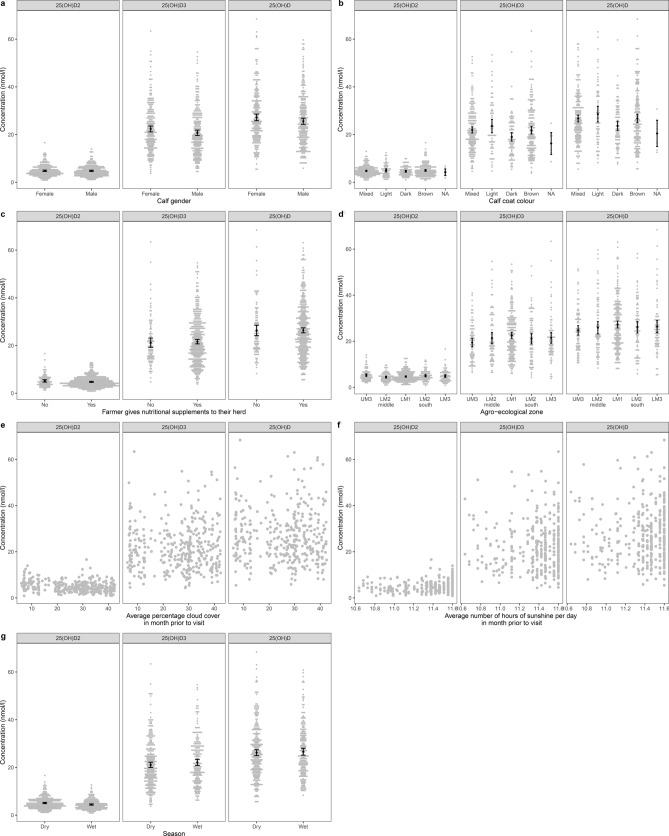
Graphs showing serum 25(OH)D concentration and calf and environmental level variables of interest. Black points represent the mean with 95% confidence interval. **(a)** Calf gender; **(b)** calf coat colour; **(c)** nutritional supplements; **(d)** agro-ecological zone; **(e)** average percentage cloud cover in month prior to visit; **(f)** average number of hours of sunshine per day in month prior to visit; **(g)** wet or dry season.

**Figure 4:**
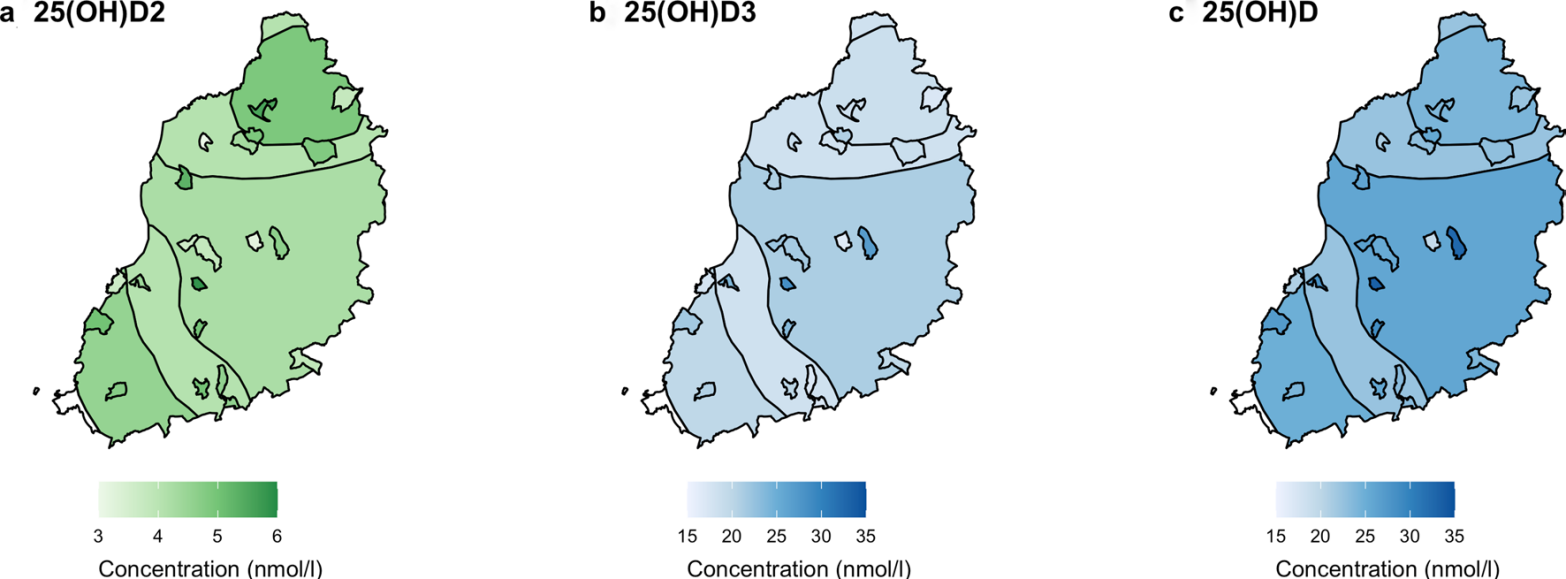
Median serum 25(OH)D concentration (nmol/l) in each agro-ecological zone and sublocation. Figures a-c represent: **(a)** 25(OH)D2; **(b)** 25(OH)D3 and **(c)** 25(OH)D. Refer to Fig. 4 for the names of the agro-ecological zones and sublocations.

**Figure 5:**
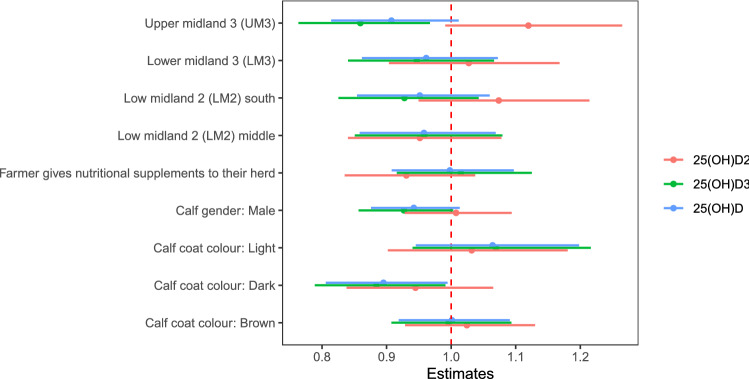
Estimates of effect sizes for the calf level factors affecting serum 25(OH)D concentration. Average model estimates are calculated for variables in the top model set (models with a cumulative Akaike weight ≤ 0.95, Supplementary Table 1 and Supplementary Fig. 2). Error bars represent the 95% confidence intervals. Factors are considered important if their confidence intervals do not span one, as indicated by the dotted vertical line.

Graphs showing serum 25(OH)D concentrations and calf level variables of interest are presented in Figs. 3 and 4 and supplementary Fig. 1. After accounting for AEZ, calves with darker coat colours had lower serum 25(OH)D_3_ and total 25(OH)D concentrations then calves with mixed coat colours (Fig. 5); whereas there was no association between calf coat colour and 25(OH)D_2_ concentration. Furthermore, calves in upper midland 3 (UM3) had lower serum concentrations of 25(OH)D_3_ then calves in low midland 1 (LM1) however there was no association between AEZ and 25(OH)D_2_ or total 25(OH)D concentrations (Fig. 5). In addition, nutritional feeding and calf gender was not associated with any of the serum 25(OH)D concentrations (Fig. 5).

### Association with environmental level factors

**Figure 6:**
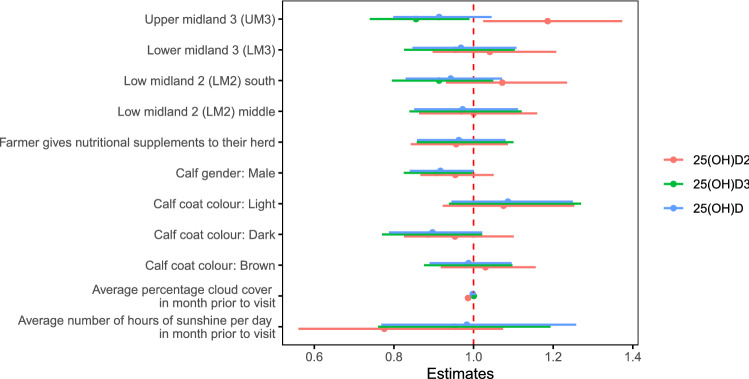
Estimates of effect sizes for the environmental level factors affecting serum 25(OH)D concentration after accounting for the calf level factors. Average model estimates are calculated for variables in the top model set (models with a cumulative Akaike weight ≤ 0.95, Supplementary Table 2 and Supplementary Fig. 3). Error bars represent the 95% confidence intervals. Factors are considered important if their confidence intervals do not span one, as indicated by the dotted vertical line.

**Figure 7:**
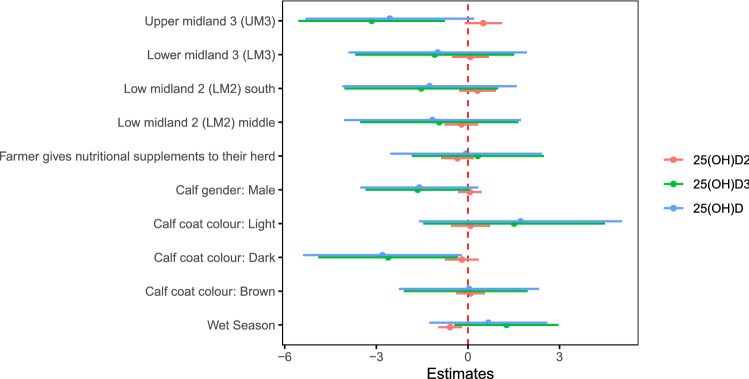
Estimates of effect sizes for the effect of wet/dry season on serum 25(OH)D concentration after accounting for the calf level factors. Error bars represent the 95% confidence intervals. Average model estimates are calculated for variables in the top model set (models with a cumulative Akaike weight ≤ 0.95, Supplementary Table 3 and Supplementary Fig. 4). Factors are considered important if their confidence intervals do not span one, as indicated by the dotted vertical line.

After accounting for calf coat colour and AEZ, there was no association between serum 25(OH)D concentration and average cloud cover or number of hours of sunshine per day in the month prior to the visit (Fig. 6). There was however an association between serum 25(OH)D_2_ concentration and season, cattle which were born during the wet season had lower serum 25(OH)D_2_ concentrations then those born during the dry season (Fig. 7).

### Association with infectious disease mortality and morbidity

Out of the 527 cattle enrolled in this study, 81 (15.4%) individuals died as a result of infectious causes before the age of 1 year. A further nine individuals died of non-infectious causes such as trauma, cassava poisoning or mis-mothering and were censored from the study. In addition, 284 (53.89%) calves experienced a clinical episode during the course of the study. The median time to first clinical illness was 106 days (interquartile range = 122 days).

**Figure 8:**
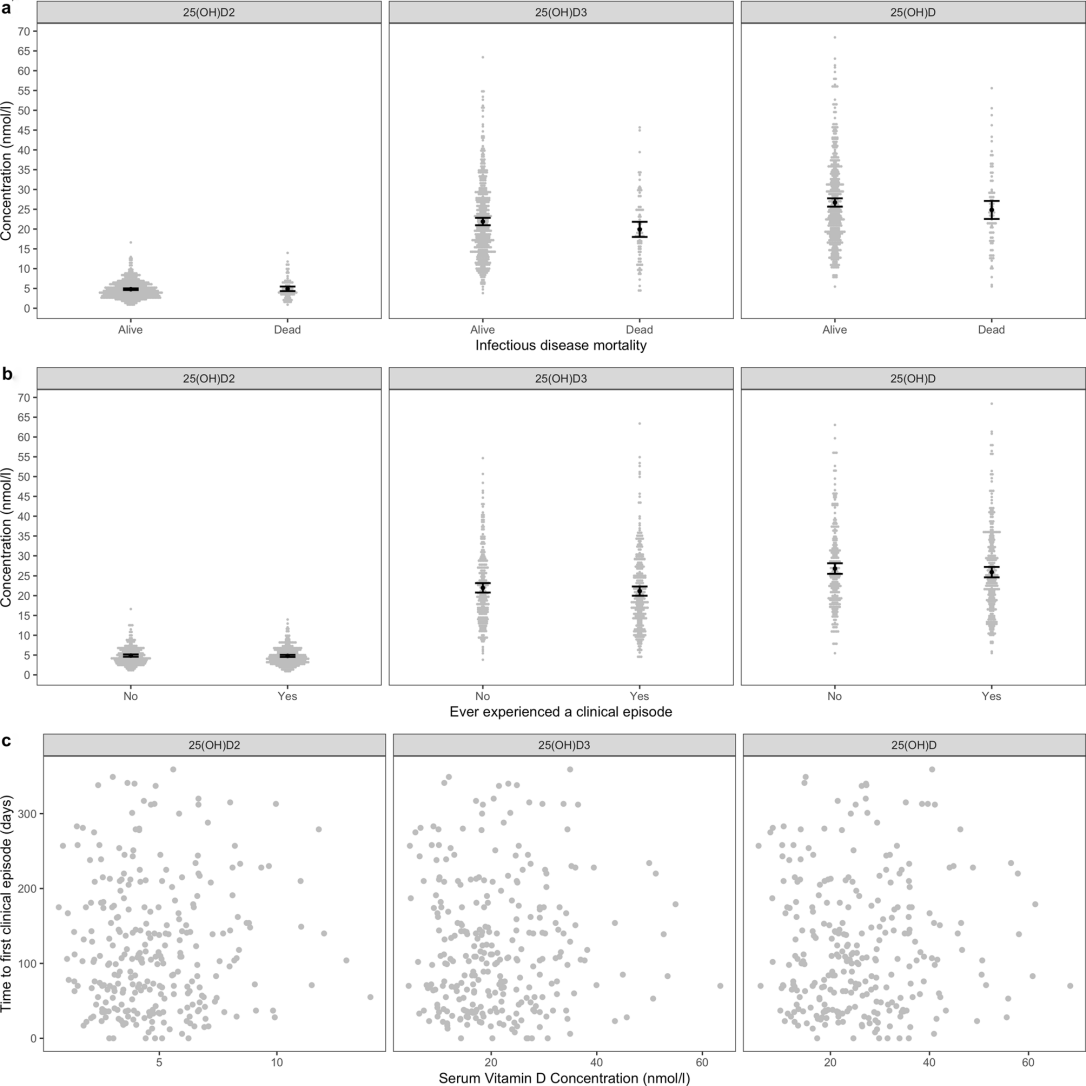
Serum 25(OH)D concentration and infectious disease mortality and morbidity by one year of age. Black points represent the mean with 95% confidence interval. **(a)** Infectious disease mortality; **(b) **experiencing a clinical episode by 51 weeks of age and **(c)** time to first clinical episode vs. serum 25(OH)D concentration.

**Figure 9:**
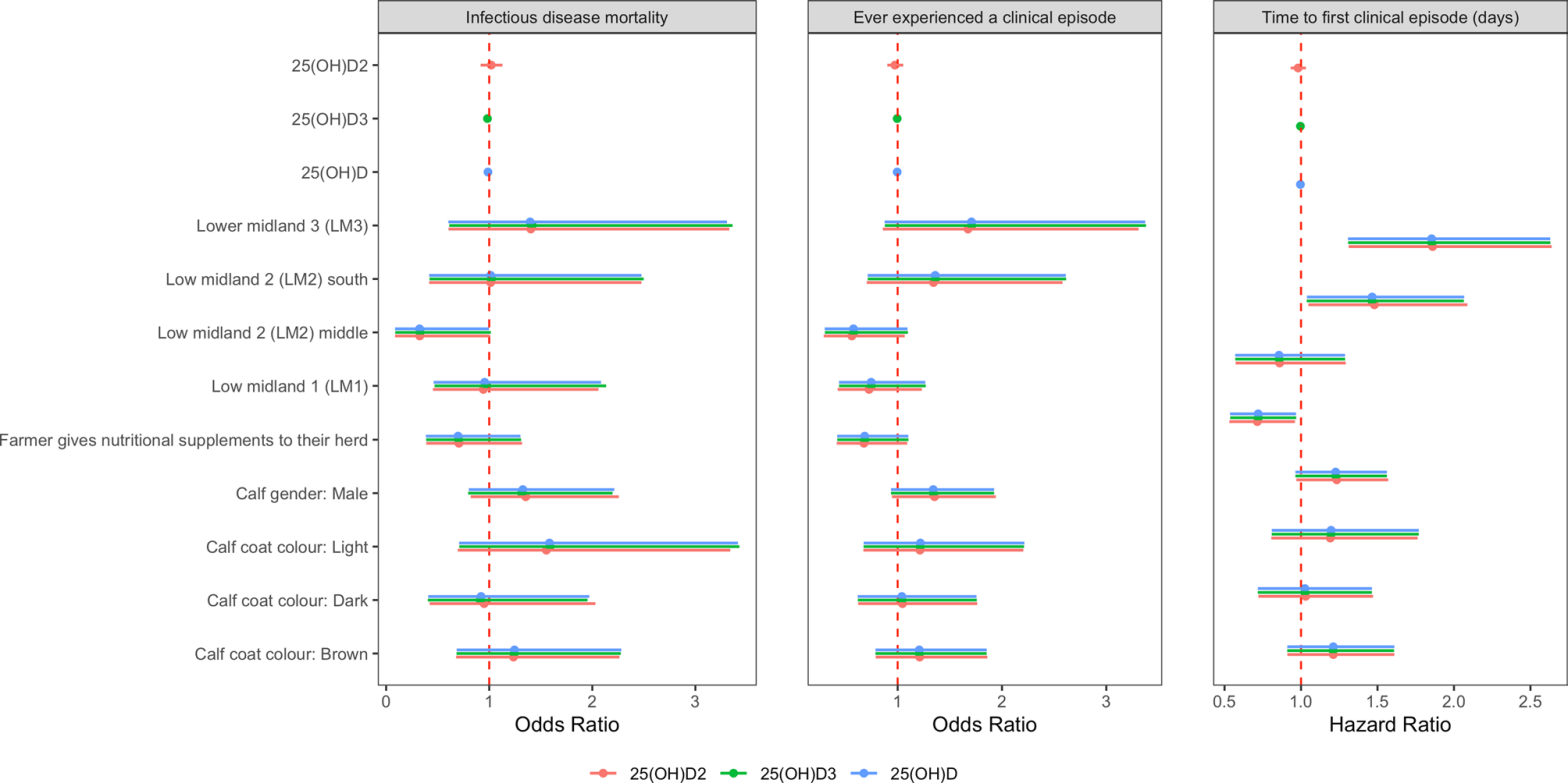
Estimates of effect sizes for the association between infectious disease mortality and morbidity by one year of age and serum 25(OH)D concentration after accounting for calf level factors and vitamin D. Factors are considered important if their confidence intervals do not span one, as indicated by the dotted vertical line.

After accounting for calf level factors and AEZ, there was no association between serum 25(OH)D concentrations and mortality from infectious disease or ever experiencing a clinical episode (Figs. 8 and 9). Furthermore, there was no association between serum 25(OH)D concentration and time to first clinical episode (Figs. 8 and 9). In addition, there was no association between serum 25(OH)D concentrations and host outcome when serum 25(OH)D concentrations were included as a categorical value based on tertiles.

### Heritability and genome wide association study of Vitamin D

**Figure 10:**
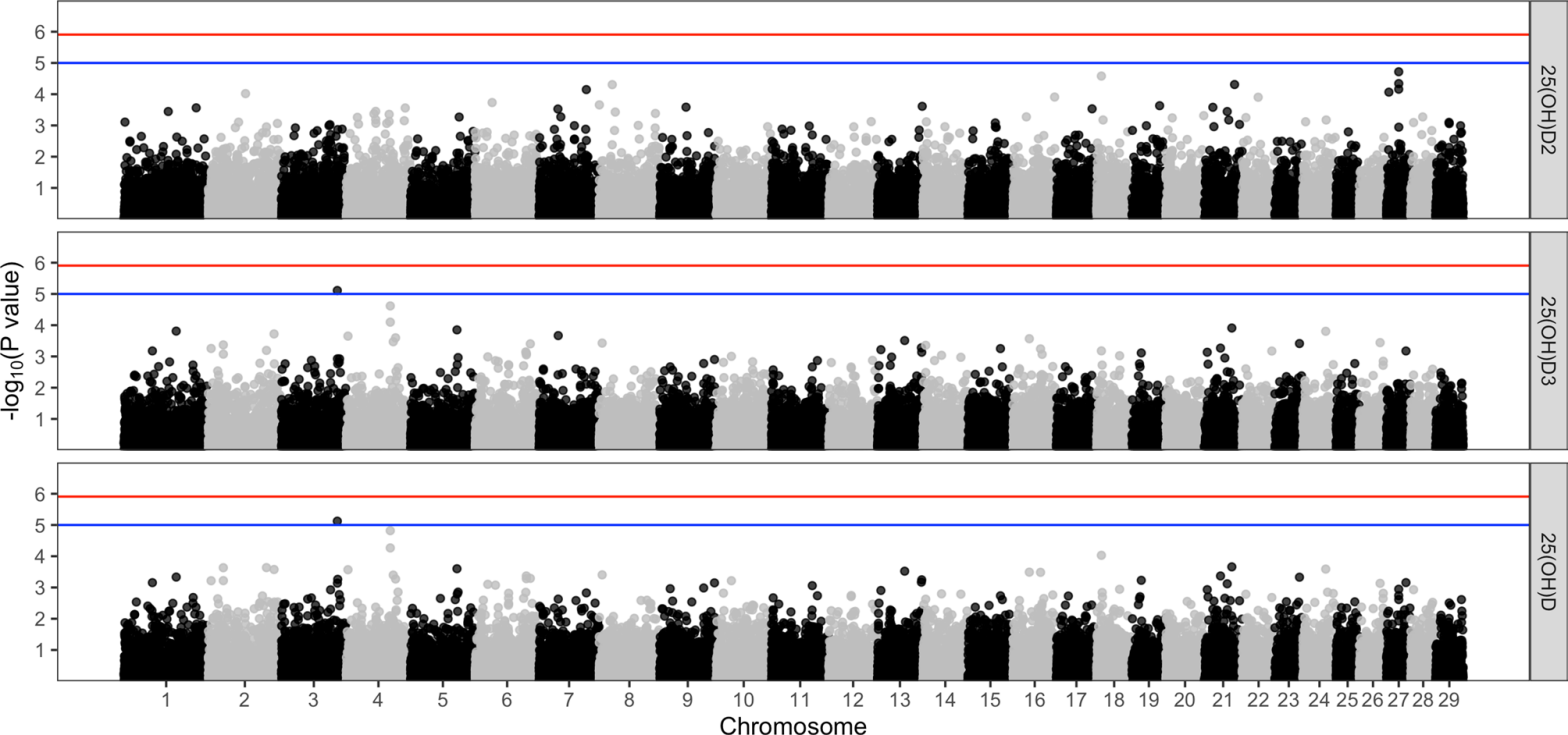
Manhattan plot of the genome wide association between SNPs and serum 25(OH)D concentration after accounting for Calf sex, calf coat colour, nutritional supplements, AEZ and the first 3 principle components as covariates. The blue line represents the suggestive significance line of < 1^–5^ and the red line represents the genome wide significance threshold, using a Bonferroni correction of < 1^–6^.

The crude heritability of vitamin D status, after accounting for confounding from calf sex, calf coat colour, nutritional supplements and AEZ, is 25(OH)D_2_ h^2^ = 62.6% (SE = 35.3); 25(OH)D_3_ h^2^ = 70.1% (SE = 30.2) and total 25(OH)D h^2^ = 71.1% (SE = 30.0), respectively (Table 2). One SNP which was associated with 25(OH)D3 and total 25(OH)D with a p-value greater than the suggestive threshold of < 1^–5^ (Fig. 10). No SNPs where associated with 25(OH)D_2_ at this level. This SNP was located at Chr 3:104814609 (Fig. 10). Ensembl Release 99 ^37^ identified one gene within ± 5000 bp of this SNP; the gene was FOXO6 which is a member of the FoxO class of transcription factors with distinct shuttling dynamics.

**Table 2:** Heritability and variance components of serum 25(OH)D concentration. The brackets represent the standard errors on the estimates.

	Heritability	V_A_	V_R_
25(OH)D2	62.6% (35.3%)	0.20 (0.17)	0.12 (0.08)
25(OH)D3	70.1% (30.2%)	0.23 (0.16)	0.10 (0.07)
25(OH)D	71.1% (30.0%)	0.20 (0.13)	0.08 (0.06)

*V*_*A*_ additive genetic variance; *V*_*R*_  residual variation.

## Discussion

Our study discovered that host factors are associated with vitamin D status in cattle. Similar to our observations in sheep in temperate regions^27,28^, we found that dark coat colour was associated with lower serum 25(OH)D_3_ concentrations in African calves which we would predict is a result of lower cutaneous production of vitamin D. Our study failed to identify clear genetic loci associated with serum 25(OH)D concentrations in cattle. This observation mirrors findings in humans which have also shown that total 25(OH)D concentration is under polygenic control with only a modest number of genetic loci linked to vitamin D status^38,39^. A GWAS study of nearly 80,000 individuals identified only six loci linked to 25(OH)D concentrations resulting in an estimate of heritability of serum 25(OH)D concentrations attributable to GWAS common SNPs at only 7.5%^38^. A more recent GWAS study of 25(OH)D concentrations involving over 400,000 participants has identified a further 63 loci but the SNP heritability of 25(OH)D was estimated to be 16.1%^39^. We only found one SNP associated with vitamin D status in this study which may reflect the relatively low coverage of the SNP array and the number of individuals in the analysis.

The lack of association between vitamin D status and mortality contrasts with cross sectional studies in humans and companion animals^11,12,21,22,24,40^. It also contrasts with some, but not all, mendelian randomisation studies which have linked increased risk of mortality in humans with genetically lower vitamin D status^10,41–43^ and with findings that vitamin D supplementation lowers mortality in patients with cancer^19^. The explanation for the discordant result in our study is unclear. A potential explanation is that vitamin D is simply a marker of ill health and a low vitamin D status is a consequence, rather than a cause, of ill-health. This concept is supported by studies which reveal that 25(OH)D concentrations decline following the development of an inflammatory response^44,45^. Similarly, cattle infected with experimental bovine diarrhoea virus experienced a significant decrease in 25(OH)D concentrations^46^. Another potential explanation is that young animals have lower vitamin D status than adults and consequently the range of 25(OH)D concentrations is smaller than in adults^27,47^. The small variation in 25(OH)D concentrations in the IDEAL calves may reduce the study’s ability to detect relationships between 25(OH)D concentrations and longer-term health outcomes. In addition, the higher infectious disease challenge faced by calves in Kenya may limit the impact of vitamin D to shape non-skeletal health^34^. Finally, 25(OH)D concentrations measured in different years tend to show little variation in adult human studies^48–52^ but neonatal vitamin D status invariably cannot absolutely predict future 25(OH)D concentrations^53^. Consequently, further studies should explore whether 25(OH)D concentrations from adult African cattle are predictive of mortality.

We found no evidence that vitamin D status was linked to subsequent infectious disease clinical episode events. This contrasts with findings in humans where low cord or neonatal serum 25(OH)D concentrations have been associated with adverse health outcomes such as early childhood lung disorders^54,55^, higher blood pressure^56^, atopic dermatitis^54^, food allergy^53^ and sepsis^57,58^. However, our finding is consistent with findings in experimental studies of respiratory infections in cattle. Calves with low vitamin D status did not have any significant differences in lung pathology following challenge with respiratory syncytical virus compared to calves with high vitamin D status^59^. Although vitamin D metabolites can dramatically influence the bovine immune response both *in-vitro*, the impact on the host immune system following *in-vivo* vitamin D supplementation is less consistent. For example, vitamin D repletion had no impact on a wide range of immune parameters in cattle^60^ and so the differences in vitamin D status in the cattle included in this study may be insufficient to modulate the immune system in a clinically detectable manner. Another potential explanation is that the calves, who resided in a tropical location, were exposed to sufficient UVB radiation to prevent the development of profound vitamin D insufficiency^61–63^. Numerous, albeit not all, studies in humans have shown that patients with the lowest vitamin D status have the greatest risk of disease development^14^ and achieve the most significant health benefits from vitamin D supplementation^64,65^.

In summary, we have shown that vitamin D status is under polygenic control in African cattle and that host factors, namely coat colour, are associated with serum 25(OH)D_3_ concentrations. We found no evidence that neonatal vitamin D status is associated with infectious diseases development or mortality in African calves. This work further illuminates the ongoing debate about the non-skeletal health benefits of vitamin D in production animals. Further studies exploring the relationship between neonatal vitamin D status and future health outcomes in non-tropical populations will be informative. In addition, studies exploring the relationship between 25(OH)D concentrations in young adult African cattle and infectious disease development and overall mortality will also further inform the discussion around the non-skeletal health benefits of vitamin D in ruminants.

## Methods

### Study population

The Infectious Disease East African Livestock (IDEAL) project was a longitudinal cohort study which was conducted in Western Kenya between 2007–2009; see Bronsvoort et al. (2013) for a detailed description of the study design ^36^. Briefly, 548 indigenous East African Shorthorn Zebu calves in were randomly selected using a stratified two-stage clustered study design. In the first stage, 20 sublocations (the smallest administrative unit in Kenya) were selected from five agro-ecological zones (AEZ), across an area of roughly 45 × 90 km^2^ surrounding the town of Busia, Fig. 1. In the second stage, around 28 calves were recruited from each sublocation. Only one calf was recruited per farm. Calves were between 3 and 7 days old at recruitment and were followed for the first year of life. They were visited every 5 weeks for a clinical examination by a team of veterinarians and animal health assistants. If the farmer reported that the calf was experiencing a clinical episode, then an interim visit was carried out. If the calf died, then a post-mortem examination was performed to attribute the cause of death. Blood and tissue samples were collected in association with all visits and screened using a range of laboratory based diagnostic methods for over 100 different pathogens or infectious exposures. Information of the dams of the calves was also collected at each 5-weekly visit until the calf was weaned. This included information on the body condition score of the dam, which was measured on a scale of 1–9 with 1–3 meaning lean, 4–6 meaning normal weight and 7–9 being fat^66^. All the data collected during the IDEAL is stored in a comprehensive open-access database, in addition, the samples collected during the IDEAL project are stored in a bio-repository at − 80 °C^67^.

### Measuring serum 25(OH)D concentration

In 2018, the serum concentration of 25(OH)D_2_ and 25(OH)D_3_ from bio-banked sampled taken from the 548 IDEAL calves at the recruitment visit (when the cattle were 3–7 days old) was measured as previously described^68^. Serum concentrations of 25(OH)D_2_ and 25(OH)D_3_ were measured by liquid chromatography tandem mass spectrometry (LC–MS/MS) by the Vitamin D Animal Laboratory (VitDAL) which has been certified as proficient by the international Vitamin D Quality Assurance Scheme (DEQAS). Briefly, 200 μL of serum and 1% BSA calibrators spiked with certified reference standards 25(OH)D_2_ and 25(OH)D_3_ (Sigma-Aldrich, UK) were spiked with equal amounts of isotopically labelled internal standards (deuterium-labeled 25(OH)D_2_ (6,19,19-d_3_-25(OH)D_2_) and carbon-13-labeled 25(OH)D_3_ (23,24,25,26,27-^13^C_5_-25(OH)D_3_ (Sigma-Aldrich) and prepared by supported liquid extraction (SLE) using 96-well plates (Biotage, UK). Samples and calibrators were then subject to derivatization with DMEQ-TAD (Abcam, UK). LC–MS/MS analysis was conducted on a Shimadzu Nexera ultra-high-performance liquid chromatography (UPLC) system (Shimadzu Corporation, Kyoto, Japan) coupled to a Sciex QTrap 6500 quadrupole mass spectrometer (AB Sciex, Warrington, England). Liquid chromatography separation was carried out using a Raptor Fluorophenyl column (2.7 µm 100 Å, LC Column 100 × 2.1 mm) (Thames Restek); the mobile phase was 2 mM ammonium formate in water with 0.1% formic acid (A) and 2 mM ammonium formate in methanol with 0.1% formic acid (B). For mass spectrometer analysis, ionization was performed by electrospray ionization (ESI) in positive ion mode and multiple reaction monitoring (MRM) was used to monitor and quantify derivatized standards and endogenous vitamin D analytes, 25(OH)D_2_ and 25(OH)D_3,_ and internal standards, d_3_-25(OH)D_2_ and ^13^C_5_-25(OH)D_3_. Total 25(OH)D is defined as the sum of 25(OH)D_2_ and 25(OH)D_3_.

### Genotyping of the cattle and SNP quality control

All the cattle where genotyped with the 50 K Illumina BovineSNP50 beadchip v.1. The beadchip contains 55,777 SNPs spread evenly throughout the genome before quality control. Quality control was applied to all SNP data prior to analysis using PLINK 2.0 ^69,70^. SNPs with a minor allele frequency of < 0.01 or a call rate of < 90% were removed. In addition, individuals with a call rate of < 90% were removed and only autosomal SNPs where included in the analysis. This left 40,405 autosome variants and 518 cattle which passed quality control checks.

### Statistical analysis

The distributions of the total 25(OH)D, 25(OH)D_2_ and 25(OH)D_3_ were examined and the difference in median concentrations of 25(OH)D_2_ and 25(OH)D_3_ within samples were tested with a paired Wilcoxon signed rank test, as well as their correlation (Spearman rank correlation). Unless otherwise stated, all analysis was carried out using R version 3.6.0 ^71^ and the figures were created using the R package *ggplot2*^72^.

### Association with calf level factors

The calf level factors associated with serum 25(OH)D concentration was investigated using multi-model inference with model averaging. Calf level factors which were initially thought to be associated with serum 25(OH)D concentration were: calf gender; dam and calf coat colour, coat pattern and hair length; calf weight at recruitment; calf rectal temperature; dam body condition score and whether or not the farmer gives nutritional supplements to their herd.

Correlation between calf level variables were assessed using Pearson’s product moment correlation for the continuous variables and chi-squared tests for the categorical variables. Since calf coat colour was correlated with variables which described both the dam’s and the calf’s coats, we avoided collinearity issues amongst the explanatory variables by only including calf coat colour in the global model to represent the coat descriptions. No other variables were correlated. Variables which were associated with any of the serum vitamin D metabolite concentrations with a P value < 0.2 was then included in the global model.

The global model for each metabolite was a generalised linear model fitted by restricted maximum likelihood (REML), with a gamma error distribution and log link function. The variables which passed the above selection criteria and were included as fixed effects in all the global model were calf gender, calf coat colour and whether or not the farmer gives nutritional supplements to their herd. Agroecological zone was included in all models as a fixed effect to account for similarity between cattle coming from the same AEZ. The global model was checked for variance inflation factors using the *vif* function of the *car* package^73^; no variables had a generalised variance inflation factor (GVIF^1/(2*df)^) greater than the threshold value of 2^74^.

Model averaging was carried out using the ‘MuMln’ package in R version 3.6.0^71,75^ on the model set generated from the global model selecting all models were the cumulative Akaike weight less than or equal to 0.95. Parameter estimates and their confidence intervals was generated from this model set. The model averaging results were similar to those results produced from models without averaging.

### Association with environmental level factors

Historic weather records for Busia, Kenya was obtained from https://www.worldweatheronline.com. Data was only available from June 2008 onwards, however the recruitment visits to the calves began in October 2007, therefore a large proportion of cattle are missing environmental data. In total we have complete records for 358 (68%) of individuals. The historic weather data consisted of number of hours of sunshine per day and percentage of cloud cover. This information was collapsed into monthly averages for the month prior to the visit at which the serum vitamin D sample was tested, to account for the weather variation the dam experienced before having the calf.

The calf level model described above, was extend to include the environmental level data. Therefore, the fixed effects included in the environmental model were: calf gender, calf coat colour and whether or not the farmer gives nutritional supplements to their herd; average total monthly sun hours prior to visit and average percentage cloud cover in month prior to visit (%). Agroecological zone was included in all models as a fixed effect to account for similarity between cattle coming from the same AEZ. The same methods of model selection, validation and averaging was used as the calf level model.

Broadly, the dry season in Kenya occurs between January to March and warm, dry period also occurs between July to October, whilst the wetter months are between April to June and November to December. The season in which the sample was taken was defined and the same modelling procedure as described above was used to evaluate the effect of the wet/dry season on serum 25(OH)D concentration. Since this broad definition of the wet/dry season does not rely on historic weather records, season could be defined for all 518 cattle.

### Association with infectious disease mortality and morbidity

To investigate the relationship between infectious disease mortality by 1 year of age and 25(OH)D concentration a generalised linear model with a binomial error distribution and logit link functions was used. Serum 25(OH)D concentration was included as a continuous fixed effect in the model along with the calf level variables (calf coat colour, calf sex, nutritional supplements) and AEZ. A separate model was built for each vitamin D measurement.

Infectious disease morbidity was measured using two methods. Firstly, it was measured as whether or not the IDEAL veterinary clinicians recorded the calf to have ever experienced a clinical episode during its enrolment in the IDEAL study. This was modelled in the same way as infectious disease mortality, with a generalised linear model with a binomial error distribution and logit link functions was used. Serum 25(OH)D concentration was fitted as a continuous fixed effect.

Secondly, infectious disease morbidity was measured as the time to when the IDEAL veterinary clinicians first recorded that a calf experienced a clinical episode. In the event that cattle died from infectious causes before experiencing a clinical episode, the date of death was counted as the time to first clinical episode as it is an extreme case of a clinical illness. The time to first clinical episode was modelled using a Cox proportional hazards regression model. Like the other infectious disease morbidity model, 25(OH)D concentration was included as a continuous fixed effect in the model along with the calf level variables (calf coat colour, calf sex, nutritional supplements) and AEZ. A separate model was built for each vitamin D measurement. The hazard ratio was used to determine the effect of one unit change in serum 25(OH)D concentration on the risk of experiencing a clinical episode.

The association with infectious disease mortality and morbidity analysis was repeated with vitamin D included as a categorical variable based on tertiles in case the relationship between serum 25(OH)D concentration and health outcomes was not linear.

### Heritability of vitamin D

To calculate the heritability of the serum 25(OH)D concentration, we first removed cattle which where related with more than a second-degree relative (corresponding to a KING kinship coefficient of 0.25) using PLINK2^69,70^. This left 494 individuals for analysis. The variation in 25(OH)D concentration was partitioned into the additive genetic and the residual variance components using generalised linear mixed models with gamma error distributions and log link functions in ASReml version 3^76^. Calf sex, calf coat colour, nutritional supplements and AEZ where included as fixed effects in the model to account for the potential confounding effects of these factors.

The variance of the additive genetic effect, V_A_ (the genetic cause of variation in 25(OH)D concentration) was estimated by including individual identity linked with the genomic-relationship matrix (GRM) as a random effect in the model. The GRM was created constructed from the inverse of the IBS matrix which was created using the R package *SNPRelate*^77^. The remaining variation, is the residual error. It was assumed that the random effect, followed a normal distribution, $a\sim N\left(0,{\sigma }_{a}^{2}G\right)$, where ${\sigma }_{a}^{2}$ is the additive genetic variation and G is the genomic relation matrix described above; the residual error $e\sim N(0,{\sigma }_{e}^{2}I)$ with residual variance ${\sigma }_{e}^{2}$ and identity matrix, I.

The narrow-sense heritability of serum 25(OH)D concentration (${h}^{2}$) was defined as the proportion of phenotypic variance explained by the additive genetic variance, ${h}^{2}= {\sigma }_{a}^{2}/({\sigma }_{a}^{2}+ {\sigma }_{e}^{2})$. It describes the extent to which differences between individuals are determined by additive genetic effects^78^. The heritability analyses were carried out in ASReml version 3.0 as previously described^76^. Note, these estimates are likely to be inflated as they do not include any population structure that may be confounded with genetic effects. It was not possible to include these in the model due to the small numbers of individuals genotyped.

### Genome wide association with serum 25(OH)D concentration

A genome wide association study (GWAS) was carried out on the quality controlled dataset using the GMMAT package in R^71,79^. Generalised linear models fitted with a gamma error distribution and log link functions was used to evaluate the association between serum 25(OH)D concentration and each SNP. Calf sex, calf coat colour, nutritional supplements, AEZ and the first three principle components where included as covariates in the model to account for population structure and confounding factors. A Bonferroni correction was applied to the genome-wide significance threshold, calculated as − log_10_(p) = − log_10_(0.05/40,405) = 5.91.p = 0.05/40,405 = 1^–6^. SNPs which were associated with vitamin D with a p-value above this threshold where considered to be of interest.

### Ethics declarations

The IDEAL project was reviewed and approved by the University of Edinburgh Ethics Committee (reference number OS 03-06) and also by the Institute Animal Care and Use Committee of the International Livestock Research Institute, Nairobi. Standard techniques were used to collect blood and faecal samples for diagnosis and identification of disease and infecting pathogens. The calves were restrained by professional animal health assistants or by qualified veterinary surgeons. A veterinary surgeon was available to examine any calf falling sick during the course of the study. Any calf that was in severe distress due to trauma or disease was humanely euthanized by intravenous injection of sodium pentobarbital by a veterinary surgeon. All participating farmers gave informed consent in their native language before recruiting their animals into the study. All methods were carried out in accordance with relevant guidelines and regulations.

## Supplementary information

Supplementary Legends.

Supplementary Figure 1.

Supplementary Figure 2.

Supplementary Figure 3.

Supplementary Figure 4.

Supplementary Table 1.

Supplementary Table 2.

Supplementary Table 3.
