# Supplementary material for: Determinants of vitamin D status in Kenyan calves
Source: Sci Rep. 2020 Nov 25;10:20590. doi: 10.1038/s41598-020-77209-5 (PMC7688966; doi:10.1038/s41598-020-77209-5)
Supplement: Supplementary file 1 — Supplementary Legends. [file 41598_2020_77209_MOESM1_ESM.docx]

# Determinants of vitamin D status in Kenyan calves

## Rebecca Callaby ^1,2^ , Emma Hurst ^3^ , Ian Handel ^1^, Phil Toye ^4^, Barend M de C Bronsvoort ^1,2^, Richard J Mellanby ^3^ *

^1^ The Epidemiology, Economics and Risk Assessment (EERA) Group, The Roslin Institute and The Royal (Dick) School of Veterinary Studies, The University of Edinburgh, Easter Bush Veterinary Centre, Roslin, Midlothian, EH25 9RG, United Kingdom

^2^ Centre for Tropical Livestock Genetics and Health (CTLGH), The Roslin Institute, University of Edinburgh, Easter Bush Campus, EH25 9RG, UK

^3^ The Vitamin D Animal Laboratory (VitDAL), The Royal (Dick) School of Veterinary Studies and The Roslin Institute, The University of Edinburgh, Easter Bush Veterinary Centre, Roslin, Midlothian, EH25 9RG, United Kingdom

^4^ International Livestock Research Institute and Centre for Tropical Livestock Genetics and Health, Nairobi, Kenya.

*correspondence to Richard.Mellanby@ed.ac.uk

## Supplementary Information

Supplementary Figure 1: Examples of calf coat colours

Supplementary Figure 2: Estimates of effect sizes for the calf level factors affecting serum 25(OH)D concentration. Each model estimates and the average model estimates are calculated for variables in the top model set (models with a cumulative Akaike weight ≤0.95). Error bars represent the 95% confidence intervals. Factors are considered important if their confidence intervals do not span one, as indicated by the dotted vertical line. This figure was created using the package ggplot2 in R version 3.6.0 (71, 72)

Supplementary Figure 3: Estimates of effect sizes for the top models with a cumulative Akaike weight ≤0.95 for the association between environmental level factors and serum 25(OH)D concentration after accounting for the calf level factors and agro-ecological zone (AEZ) as fixed effects. Error bars represent the 95% confidence intervals. Factors are considered important if their confidence intervals do not span zero. This figure was created using the package ggplot2 in R version 3.6.0 (71, 72)

Supplementary Figure 4: Estimates of effect sizes for the top models with a cumulative Akaike weight ≤0.95 for the association between wet/dry season and serum 25(OH)D concentration after accounting for the calf level factors and agro-ecological zone (AEZ) as fixed effects. Error bars represent the 95% confidence intervals. Factors are considered important if their confidence intervals do not span zero. This figure was created using the package ggplot2 in R version 3.6.0 (71, 72)

Supplementary Table 1: All model considered for the association between calf level factors and serum 25(OH)D concentration. Only the top models with cumulative Akaike weight ≤0.95 were included in the model averaging presented in this manuscript. Each row in the table represents a linear mixed model, with + indicating the inclusion of a given variable as a fixed effect within the model. Agro-ecological zone (AEZ) was included as fixed effects in all models. R^2^ and adjusted R^2^, degrees of freedom (df), ΔAIC_C_, model weight (ω_i_), cumulative Akaike weight (cω_i_), are present for each model.

Supplementary Table 2: All model considered for the association between environmental level factors and serum 25(OH)D concentration. Calf level factors and agro-ecological zone (AEZ) are also included in the model as fixed effects. Only the top models with cumulative Akaike weight ≤0.95 were included in the model averaging presented in this manuscript. Each row in the table represents a linear mixed model, with + indicating the inclusion of a given variable as a fixed effect within the model. Agro-ecological zone (AEZ) was included as fixed effects in all models. R^2^ and adjusted R^2^, degrees of freedom (df), ΔAIC_C_, model weight (ω_i_), cumulative Akaike weight (cω_i_), are present for each model.

Supplementary Table 3: All model considered for the association between wet/dry season and serum 25(OH)D concentration. Calf level factors and agro-ecological zone (AEZ) are also included in the model as fixed effects. Only the top models with cumulative Akaike weight ≤0.95 were included in the model averaging presented in this manuscript. Each row in the table represents a linear mixed model, with + indicating the inclusion of a given variable as a fixed effect within the model. Agro-ecological zone (AEZ) was included as fixed effects in all models. R^2^ and adjusted R^2^, degrees of freedom (df), ΔAIC_C_, model weight (ω_i_), cumulative Akaike weight (cω_i_), are present for each model.
