# Supplementary figures and images for: Determinants of vitamin D status in Kenyan calves
Source: Sci Rep. 2020 Nov 25;10:20590. doi: 10.1038/s41598-020-77209-5 (PMC7688966; doi:10.1038/s41598-020-77209-5)

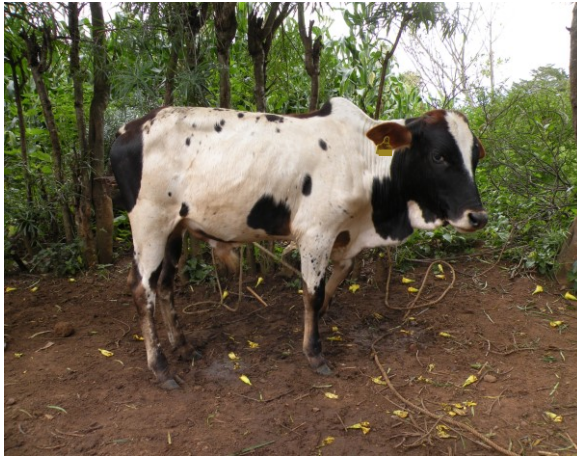

a) Mixed

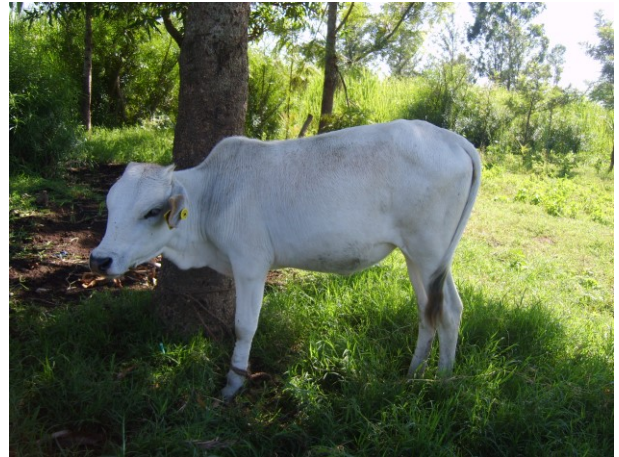

b) Light

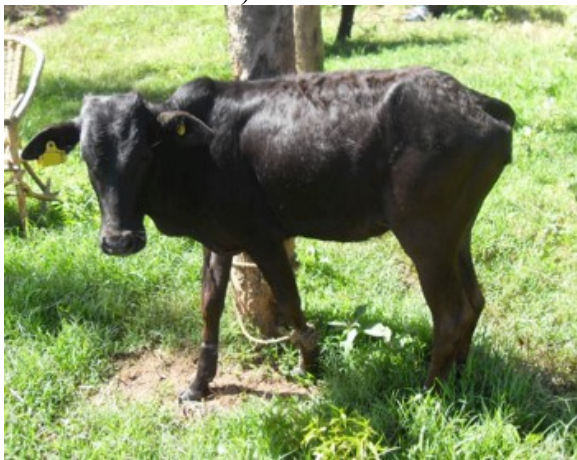

c) Dark

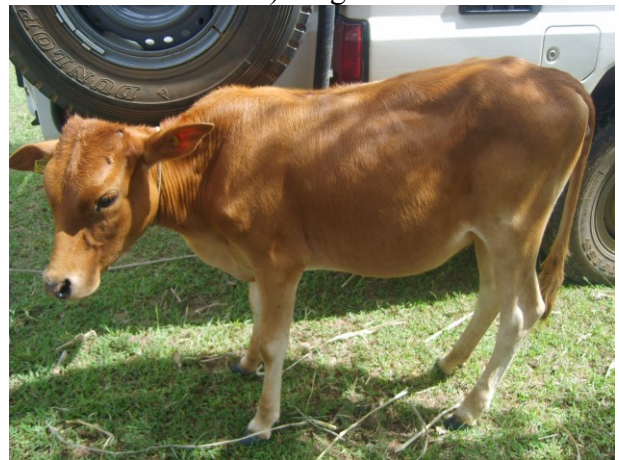

d) Brown

Supplement: Supplementary file 2 — Supplementary Figure 1. [file 41598_2020_77209_MOESM2_ESM.pdf]

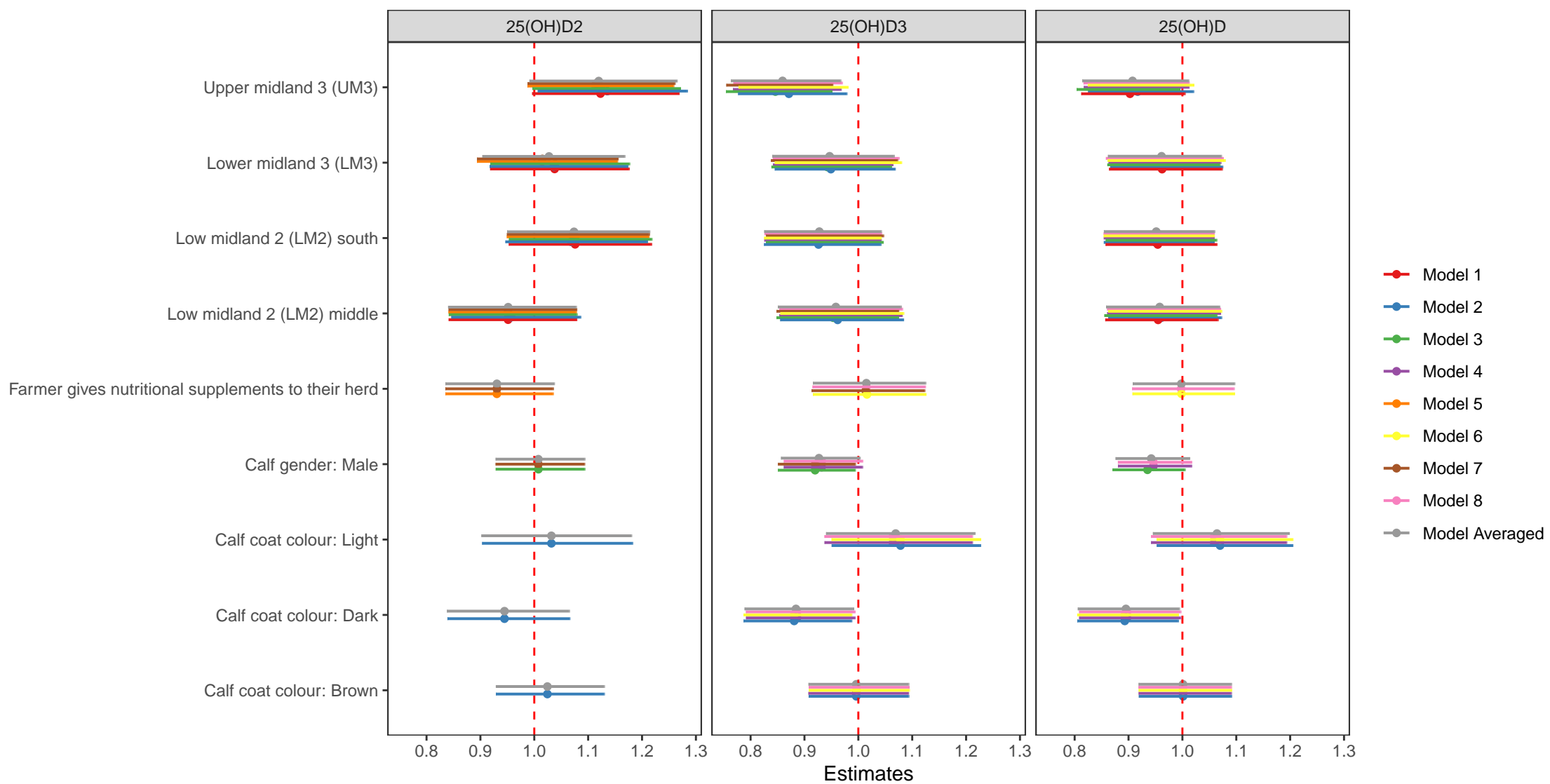

Supplement: Supplementary file 3 — Supplementary Figure 2. [file 41598_2020_77209_MOESM3_ESM.pdf]

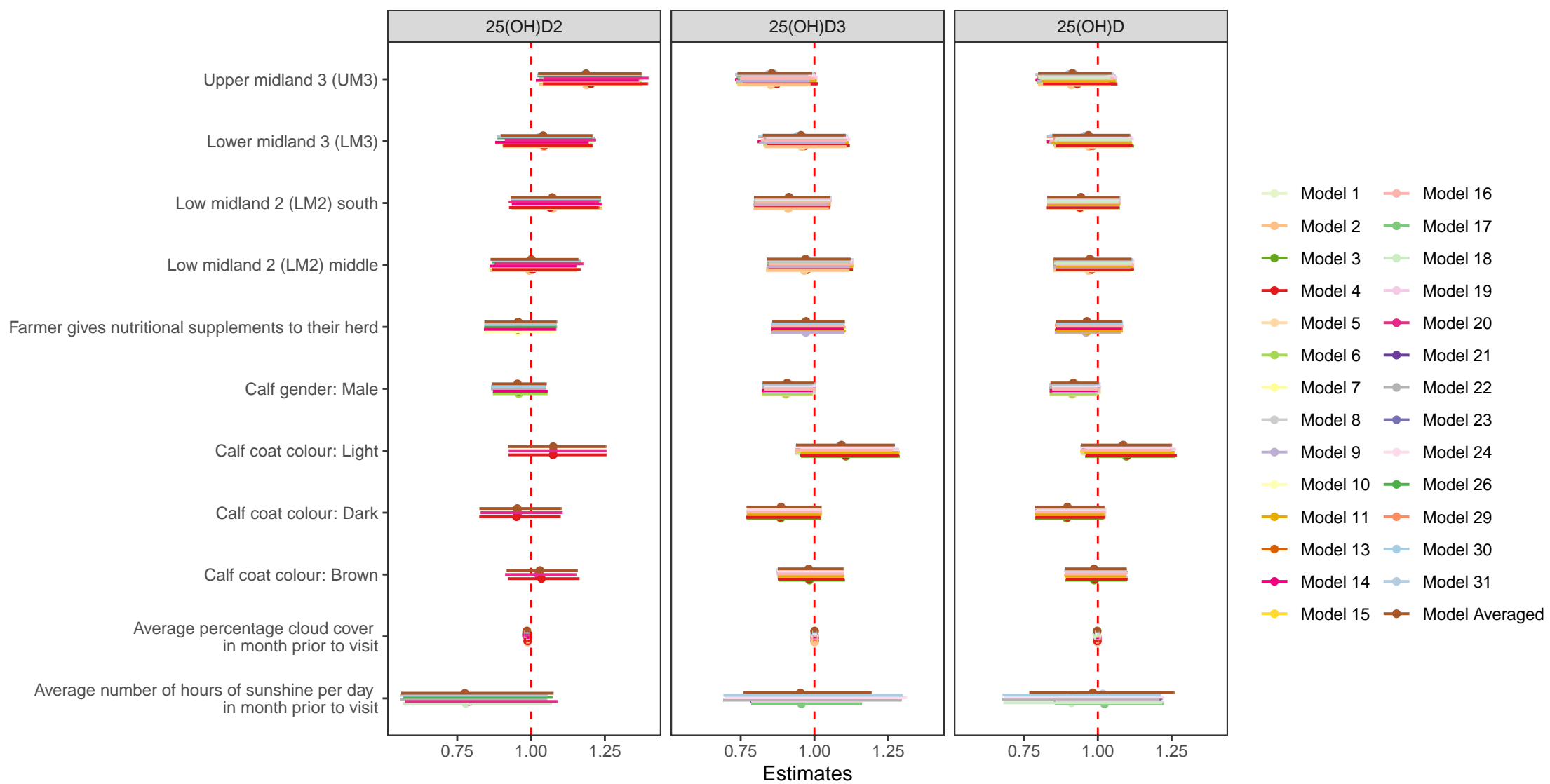

Supplement: Supplementary file 4 — Supplementary Figure 3. [file 41598_2020_77209_MOESM4_ESM.pdf]

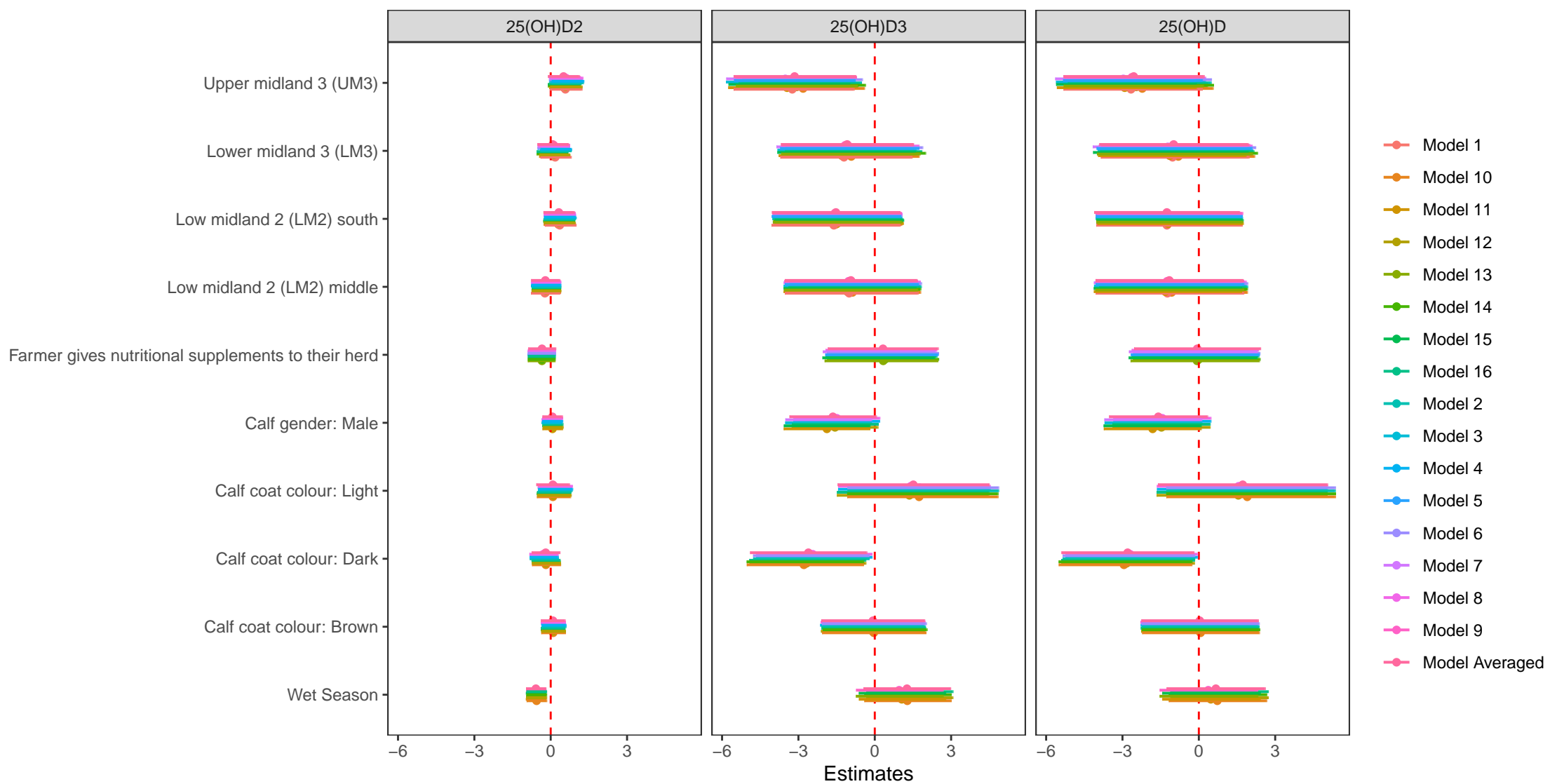

Supplement: Supplementary file 5 — Supplementary Figure 4. [file 41598_2020_77209_MOESM5_ESM.pdf]
