# Supplementary material for: Determinants of vitamin D status in Kenyan calves
Source: Sci Rep. 2020 Nov 25;10:20590. doi: 10.1038/s41598-020-77209-5 (PMC7688966; doi:10.1038/s41598-020-77209-5)
Supplement: Supplementary file 6 — Supplementary Table 1. [file 41598_2020_77209_MOESM6_ESM.docx]

| **Metabolite** | **Model** | **Intercept** | **AEZ** | **Calf coat colour** | **Calf gender** | **Nutritional supplements use** | ***R^2^*** | ***Adjusted R^2^*** | **df** | **ΔAIC_C_** | **ω_i_** | **cω_i_** |
| --- | --- | --- | --- | --- | --- | --- | --- | --- | --- | --- | --- | --- |
| 25(OH)D2 | 1 | 1.55 | + | NA | NA | NA | 0.01 | 0.01 | 6 | 0.00 | 0.35 | 0.35 |
|  | 5 | 1.61 | + | NA | NA | + | 0.02 | 0.02 | 7 | 0.23 | 0.31 | 0.65 |
|  | 3 | 1.54 | + | NA | + | NA | 0.01 | 0.01 | 7 | 2.02 | 0.13 | 0.78 |
|  | 7 | 1.61 | + | NA | + | + | 0.02 | 0.02 | 8 | 2.26 | 0.11 | 0.89 |
|  | 2 | 1.54 | + | + | NA | NA | 0.02 | 0.02 | 9 | 4.20 | 0.04 | 0.93 |
|  | 6 | 1.61 | + | + | NA | + | 0.02 | 0.02 | 10 | 4.53 | 0.04 | 0.97 |
|  | 4 | 1.53 | + | + | + | NA | 0.02 | 0.02 | 10 | 6.17 | 0.02 | 0.99 |
|  | 8 | 1.60 | + | + | + | + | 0.02 | 0.02 | 11 | 6.51 | 0.01 | 1.00 |
| 25(OH)D3 | 4 | 3.17 | + | + | + | NA | 0.04 | 0.04 | 10 | 0.00 | 0.30 | 0.30 |
|  | 3 | 3.17 | + | NA | + | NA | 0.02 | 0.02 | 7 | 0.76 | 0.20 | 0.50 |
|  | 2 | 3.13 | + | + | NA | NA | 0.03 | 0.03 | 9 | 1.13 | 0.17 | 0.67 |
|  | 8 | 3.16 | + | + | + | + | 0.04 | 0.04 | 11 | 2.00 | 0.11 | 0.78 |
|  | 7 | 3.16 | + | NA | + | + | 0.02 | 0.02 | 8 | 2.76 | 0.08 | 0.86 |
|  | 6 | 3.12 | + | + | NA | + | 0.03 | 0.03 | 10 | 3.12 | 0.06 | 0.92 |
|  | 1 | 3.12 | + | NA | NA | NA | 0.01 | 0.01 | 6 | 3.25 | 0.06 | 0.98 |
|  | 5 | 3.11 | + | NA | NA | + | 0.01 | 0.01 | 7 | 5.22 | 0.02 | 1.00 |
| 25(OH)D | 4 | 3.35 | + | + | + | NA | 0.03 | 0.03 | 10 | 0.00 | 0.24 | 0.24 |
|  | 2 | 3.32 | + | + | NA | NA | 0.02 | 0.02 | 9 | 0.21 | 0.21 | 0.45 |
|  | 3 | 3.35 | + | NA | + | NA | 0.01 | 0.01 | 7 | 0.48 | 0.19 | 0.64 |
|  | 1 | 3.31 | + | NA | NA | NA | 0.01 | 0.01 | 6 | 1.82 | 0.10 | 0.74 |
|  | 8 | 3.35 | + | + | + | + | 0.03 | 0.03 | 11 | 2.09 | 0.08 | 0.82 |
|  | 6 | 3.32 | + | + | NA | + | 0.02 | 0.02 | 10 | 2.29 | 0.08 | 0.90 |
|  | 7 | 3.35 | + | NA | + | + | 0.01 | 0.01 | 8 | 2.54 | 0.07 | 0.97 |
|  | 5 | 3.31 | + | NA | NA | + | 0.01 | 0.01 | 7 | 3.87 | 0.03 | 1.00 |
