# Supplementary material for: Determinants of vitamin D status in Kenyan calves
Source: Sci Rep. 2020 Nov 25;10:20590. doi: 10.1038/s41598-020-77209-5 (PMC7688966; doi:10.1038/s41598-020-77209-5)
Supplement: Supplementary file 7 — Supplementary Table 2. [file 41598_2020_77209_MOESM7_ESM.docx]

| **Metabolite** | **Model** | **Intercept** | **AEZ** | **Average Cloud Cover (%)** | **Average number of hours of sunshine** | **Calf coat colour** | **Calf gender** | **Nutritional supplements use** | ***R^2^*** | ***Adjusted R^2^*** | **df** | **ΔAIC_C_** | **ω_i_** | **cω_i_** |
| --- | --- | --- | --- | --- | --- | --- | --- | --- | --- | --- | --- | --- | --- | --- |
| 25(OH)D2 | 18 | 4.79 | + | -0.02 | -0.25 | NA | NA | NA | 0.10 | 0.10 | 8 | 0.00 | 0.22 | 0.22 |
|  | 2 | 1.84 | + | -0.01 | NA | NA | NA | NA | 0.10 | 0.10 | 7 | 0.48 | 0.17 | 0.39 |
|  | 22 | 5.00 | + | -0.02 | -0.27 | NA | + | NA | 0.11 | 0.11 | 9 | 0.99 | 0.13 | 0.53 |
|  | 26 | 4.80 | + | -0.02 | -0.25 | NA | NA | + | 0.10 | 0.10 | 9 | 1.61 | 0.10 | 0.63 |
|  | 6 | 1.86 | + | -0.01 | NA | NA | + | NA | 0.10 | 0.10 | 8 | 1.76 | 0.09 | 0.72 |
|  | 10 | 1.88 | + | -0.01 | NA | NA | NA | + | 0.10 | 0.10 | 8 | 2.04 | 0.08 | 0.80 |
|  | 30 | 5.01 | + | -0.02 | -0.26 | NA | + | + | 0.11 | 0.11 | 10 | 2.61 | 0.06 | 0.86 |
|  | 14 | 1.90 | + | -0.01 | NA | NA | + | + | 0.10 | 0.10 | 9 | 3.33 | 0.04 | 0.90 |
|  | 20 | 4.60 | + | -0.02 | -0.23 | + | NA | NA | 0.11 | 0.11 | 11 | 4.40 | 0.02 | 0.92 |
|  | 4 | 1.83 | + | -0.01 | NA | + | NA | NA | 0.10 | 0.10 | 10 | 4.50 | 0.02 | 0.95 |
|  | 24 | 4.79 | + | -0.02 | -0.25 | + | + | NA | 0.11 | 0.11 | 12 | 5.70 | 0.01 | 0.96 |
|  | 8 | 1.85 | + | -0.01 | NA | + | + | NA | 0.10 | 0.10 | 11 | 6.03 | 0.01 | 0.97 |
|  | 28 | 4.63 | + | -0.02 | -0.23 | + | NA | + | 0.11 | 0.11 | 12 | 6.20 | 0.01 | 0.98 |
|  | 12 | 1.86 | + | -0.01 | NA | + | NA | + | 0.10 | 0.10 | 11 | 6.28 | 0.01 | 0.99 |
|  | 32 | 4.81 | + | -0.02 | -0.25 | + | + | + | 0.11 | 0.11 | 13 | 7.50 | 0.01 | 1.00 |
|  | 16 | 1.88 | + | -0.01 | NA | + | + | + | 0.10 | 0.11 | 12 | 7.81 | 0.00 | 1.00 |
|  | 17 | -2.05 | + | NA | 0.32 | NA | NA | NA | 0.05 | 0.05 | 7 | 18.47 | 0.00 | 1.00 |
|  | 25 | -1.91 | + | NA | 0.31 | NA | NA | + | 0.05 | 0.05 | 8 | 19.48 | 0.00 | 1.00 |
|  | 21 | -1.98 | + | NA | 0.31 | NA | + | NA | 0.05 | 0.05 | 8 | 20.04 | 0.00 | 1.00 |
|  | 29 | -1.84 | + | NA | 0.30 | NA | + | + | 0.05 | 0.05 | 9 | 21.07 | 0.00 | 1.00 |
|  | 19 | -2.20 | + | NA | 0.33 | + | NA | NA | 0.06 | 0.06 | 10 | 22.09 | 0.00 | 1.00 |
|  | 27 | -2.06 | + | NA | 0.32 | + | NA | + | 0.06 | 0.06 | 11 | 23.44 | 0.00 | 1.00 |
|  | 23 | -2.14 | + | NA | 0.32 | + | + | NA | 0.06 | 0.06 | 11 | 23.86 | 0.00 | 1.00 |
|  | 31 | -2.01 | + | NA | 0.32 | + | + | + | 0.06 | 0.06 | 12 | 25.22 | 0.00 | 1.00 |
|  | 1 | 1.54 | + | NA | NA | NA | NA | NA | 0.02 | 0.02 | 6 | 26.84 | 0.00 | 1.00 |
|  | 9 | 1.61 | + | NA | NA | NA | NA | + | 0.03 | 0.03 | 7 | 27.34 | 0.00 | 1.00 |
|  | 5 | 1.57 | + | NA | NA | NA | + | NA | 0.02 | 0.02 | 7 | 28.13 | 0.00 | 1.00 |
|  | 13 | 1.64 | + | NA | NA | NA | + | + | 0.03 | 0.03 | 8 | 28.65 | 0.00 | 1.00 |
|  | 3 | 1.54 | + | NA | NA | + | NA | NA | 0.03 | 0.03 | 9 | 31.06 | 0.00 | 1.00 |
|  | 11 | 1.61 | + | NA | NA | + | NA | + | 0.03 | 0.03 | 10 | 31.85 | 0.00 | 1.00 |
|  | 7 | 1.56 | + | NA | NA | + | + | NA | 0.03 | 0.03 | 10 | 32.58 | 0.00 | 1.00 |
|  | 15 | 1.63 | + | NA | NA | + | + | + | 0.03 | 0.03 | 11 | 33.37 | 0.00 | 1.00 |
| 25(OH)D3 | 5 | 3.18 | + | NA | NA | NA | + | NA | 0.03 | 0.03 | 7 | 0.00 | 0.17 | 0.17 |
|  | 7 | 3.18 | + | NA | NA | + | + | NA | 0.04 | 0.04 | 10 | 0.82 | 0.11 | 0.28 |
|  | 21 | 3.73 | + | NA | -0.05 | NA | + | NA | 0.03 | 0.03 | 8 | 1.84 | 0.07 | 0.35 |
|  | 13 | 3.21 | + | NA | NA | NA | + | + | 0.03 | 0.03 | 8 | 1.86 | 0.07 | 0.41 |
|  | 6 | 3.16 | + | 0.00 | NA | NA | + | NA | 0.03 | 0.03 | 8 | 1.95 | 0.06 | 0.48 |
|  | 3 | 3.13 | + | NA | NA | + | NA | NA | 0.03 | 0.03 | 9 | 2.38 | 0.05 | 0.53 |
|  | 1 | 3.12 | + | NA | NA | NA | NA | NA | 0.02 | 0.02 | 6 | 2.62 | 0.05 | 0.58 |
|  | 23 | 3.74 | + | NA | -0.05 | + | + | NA | 0.04 | 0.04 | 11 | 2.69 | 0.04 | 0.62 |
|  | 8 | 3.16 | + | 0.00 | NA | + | + | NA | 0.04 | 0.04 | 11 | 2.77 | 0.04 | 0.66 |
|  | 15 | 3.20 | + | NA | NA | + | + | + | 0.04 | 0.04 | 11 | 2.78 | 0.04 | 0.70 |
|  | 29 | 3.79 | + | NA | -0.05 | NA | + | + | 0.03 | 0.03 | 9 | 3.69 | 0.03 | 0.73 |
|  | 14 | 3.18 | + | 0.00 | NA | NA | + | + | 0.03 | 0.03 | 9 | 3.80 | 0.03 | 0.76 |
|  | 22 | 3.80 | + | 0.00 | -0.05 | NA | + | NA | 0.03 | 0.03 | 9 | 3.94 | 0.02 | 0.78 |
|  | 19 | 3.63 | + | NA | -0.04 | + | NA | NA | 0.03 | 0.03 | 10 | 4.29 | 0.02 | 0.80 |
|  | 11 | 3.15 | + | NA | NA | + | NA | + | 0.03 | 0.03 | 10 | 4.33 | 0.02 | 0.82 |
|  | 4 | 3.11 | + | 0.00 | NA | + | NA | NA | 0.03 | 0.03 | 10 | 4.35 | 0.02 | 0.84 |
|  | 9 | 3.15 | + | NA | NA | NA | NA | + | 0.02 | 0.02 | 7 | 4.48 | 0.02 | 0.86 |
|  | 17 | 3.63 | + | NA | -0.04 | NA | NA | NA | 0.02 | 0.02 | 7 | 4.49 | 0.02 | 0.87 |
|  | 2 | 3.11 | + | 0.00 | NA | NA | NA | NA | 0.02 | 0.02 | 7 | 4.59 | 0.02 | 0.89 |
|  | 31 | 3.79 | + | NA | -0.05 | + | + | + | 0.04 | 0.04 | 12 | 4.64 | 0.02 | 0.91 |
|  | 16 | 3.18 | + | 0.00 | NA | + | + | + | 0.04 | 0.04 | 12 | 4.70 | 0.02 | 0.92 |
|  | 24 | 3.66 | + | 0.00 | -0.04 | + | + | NA | 0.04 | 0.04 | 12 | 4.83 | 0.02 | 0.94 |
|  | 30 | 3.79 | + | 0.00 | -0.05 | NA | + | + | 0.03 | 0.03 | 10 | 5.80 | 0.01 | 0.95 |
|  | 27 | 3.68 | + | NA | -0.05 | + | NA | + | 0.03 | 0.03 | 11 | 6.23 | 0.01 | 0.96 |
|  | 12 | 3.13 | + | 0.00 | NA | + | NA | + | 0.03 | 0.03 | 11 | 6.29 | 0.01 | 0.96 |
|  | 25 | 3.69 | + | NA | -0.05 | NA | NA | + | 0.02 | 0.02 | 8 | 6.34 | 0.01 | 0.97 |
|  | 20 | 3.58 | + | 0.00 | -0.04 | + | NA | NA | 0.03 | 0.03 | 11 | 6.42 | 0.01 | 0.98 |
|  | 10 | 3.13 | + | 0.00 | NA | NA | NA | + | 0.02 | 0.02 | 8 | 6.43 | 0.01 | 0.98 |
|  | 18 | 3.71 | + | 0.00 | -0.05 | NA | NA | NA | 0.02 | 0.02 | 8 | 6.58 | 0.01 | 0.99 |
|  | 32 | 3.67 | + | 0.00 | -0.04 | + | + | + | 0.04 | 0.04 | 13 | 6.79 | 0.01 | 0.99 |
|  | 28 | 3.58 | + | 0.00 | -0.04 | + | NA | + | 0.03 | 0.03 | 12 | 8.37 | 0.00 | 1.00 |
|  | 26 | 3.71 | + | 0.00 | -0.05 | NA | NA | + | 0.02 | 0.02 | 9 | 8.44 | 0.00 | 1.00 |
| 25(OH)D | 5 | 3.36 | + | NA | NA | NA | + | NA | 0.02 | 0.02 | 7 | 0.00 | 0.15 | 0.15 |
|  | 7 | 3.36 | + | NA | NA | + | + | NA | 0.03 | 0.03 | 10 | 0.93 | 0.09 | 0.24 |
|  | 6 | 3.40 | + | 0.00 | NA | NA | + | NA | 0.02 | 0.02 | 8 | 1.46 | 0.07 | 0.32 |
|  | 13 | 3.40 | + | NA | NA | NA | + | + | 0.02 | 0.02 | 8 | 1.60 | 0.07 | 0.38 |
|  | 21 | 3.16 | + | NA | 0.02 | NA | + | NA | 0.02 | 0.02 | 8 | 2.05 | 0.05 | 0.44 |
|  | 3 | 3.31 | + | NA | NA | + | NA | NA | 0.02 | 0.02 | 9 | 2.20 | 0.05 | 0.49 |
|  | 1 | 3.31 | + | NA | NA | NA | NA | NA | 0.01 | 0.01 | 6 | 2.27 | 0.05 | 0.54 |
|  | 8 | 3.40 | + | 0.00 | NA | + | + | NA | 0.03 | 0.03 | 11 | 2.49 | 0.04 | 0.58 |
|  | 15 | 3.39 | + | NA | NA | + | + | + | 0.03 | 0.03 | 11 | 2.67 | 0.04 | 0.62 |
|  | 23 | 3.13 | + | NA | 0.02 | + | + | NA | 0.03 | 0.03 | 11 | 3.01 | 0.03 | 0.65 |
|  | 22 | 4.59 | + | 0.00 | -0.10 | NA | + | NA | 0.02 | 0.02 | 9 | 3.08 | 0.03 | 0.69 |
|  | 14 | 3.43 | + | 0.00 | NA | NA | + | + | 0.02 | 0.02 | 9 | 3.15 | 0.03 | 0.72 |
|  | 2 | 3.35 | + | 0.00 | NA | NA | NA | NA | 0.01 | 0.01 | 7 | 3.67 | 0.02 | 0.74 |
|  | 29 | 3.23 | + | NA | 0.01 | NA | + | + | 0.02 | 0.02 | 9 | 3.68 | 0.02 | 0.76 |
|  | 4 | 3.35 | + | 0.00 | NA | + | NA | NA | 0.03 | 0.03 | 10 | 3.69 | 0.02 | 0.79 |
|  | 9 | 3.35 | + | NA | NA | NA | NA | + | 0.01 | 0.01 | 7 | 3.86 | 0.02 | 0.81 |
|  | 11 | 3.34 | + | NA | NA | + | NA | + | 0.02 | 0.02 | 10 | 3.94 | 0.02 | 0.83 |
|  | 19 | 3.03 | + | NA | 0.02 | + | NA | NA | 0.02 | 0.02 | 10 | 4.24 | 0.02 | 0.85 |
|  | 24 | 4.44 | + | 0.00 | -0.09 | + | + | NA | 0.04 | 0.04 | 12 | 4.26 | 0.02 | 0.87 |
|  | 17 | 3.05 | + | NA | 0.02 | NA | NA | NA | 0.01 | 0.01 | 7 | 4.28 | 0.02 | 0.89 |
|  | 16 | 3.42 | + | 0.00 | NA | + | + | + | 0.04 | 0.04 | 12 | 4.32 | 0.02 | 0.90 |
|  | 31 | 3.20 | + | NA | 0.02 | + | + | + | 0.03 | 0.03 | 12 | 4.78 | 0.01 | 0.92 |
|  | 30 | 4.58 | + | 0.00 | -0.10 | NA | + | + | 0.02 | 0.02 | 10 | 4.82 | 0.01 | 0.93 |
|  | 18 | 4.46 | + | 0.00 | -0.09 | NA | NA | NA | 0.01 | 0.01 | 8 | 5.35 | 0.01 | 0.94 |
|  | 10 | 3.38 | + | 0.00 | NA | NA | NA | + | 0.01 | 0.01 | 8 | 5.37 | 0.01 | 0.95 |
|  | 20 | 4.32 | + | 0.00 | -0.08 | + | NA | NA | 0.03 | 0.03 | 11 | 5.50 | 0.01 | 0.96 |
|  | 12 | 3.38 | + | 0.00 | NA | + | NA | + | 0.03 | 0.03 | 11 | 5.52 | 0.01 | 0.97 |
|  | 25 | 3.13 | + | NA | 0.02 | NA | NA | + | 0.01 | 0.01 | 8 | 5.90 | 0.01 | 0.98 |
|  | 27 | 3.10 | + | NA | 0.02 | + | NA | + | 0.02 | 0.02 | 11 | 6.01 | 0.01 | 0.98 |
|  | 32 | 4.46 | + | 0.00 | -0.09 | + | + | + | 0.04 | 0.04 | 13 | 6.11 | 0.01 | 0.99 |
|  | 26 | 4.45 | + | 0.00 | -0.09 | NA | NA | + | 0.01 | 0.01 | 9 | 7.08 | 0.00 | 1.00 |
|  | 28 | 4.33 | + | 0.00 | -0.08 | + | NA | + | 0.03 | 0.03 | 12 | 7.35 | 0.00 | 1.00 |
