# Supplementary material for: Determinants of vitamin D status in Kenyan calves
Source: Sci Rep. 2020 Nov 25;10:20590. doi: 10.1038/s41598-020-77209-5 (PMC7688966; doi:10.1038/s41598-020-77209-5)
Supplement: Supplementary file 8 — Supplementary Table 3. [file 41598_2020_77209_MOESM8_ESM.docx]

| **Metabolite** | **Model** | **Intercept** | **AEZ** | **Season** | **Calf coat colour** | **Calf gender** | **Nutritional supplements use** | ***R^2^*** | ***Adjusted R^2^*** | **df** | **ΔAIC_C_** | **ω_i_** | **cω_i_** |
| --- | --- | --- | --- | --- | --- | --- | --- | --- | --- | --- | --- | --- | --- |
| 25(OH)D2 | 9 | 4.96 | + | + | NA | NA | NA | 0.03 | 0.03 | 7 | 0.00 | 0.35 | 0.35 |
|  | 13 | 5.27 | + | + | NA | NA | + | 0.03 | 0.03 | 8 | 0.29 | 0.30 | 0.65 |
|  | 11 | 4.92 | + | + | NA | + | NA | 0.03 | 0.03 | 8 | 1.94 | 0.13 | 0.79 |
|  | 15 | 5.23 | + | + | NA | + | + | 0.03 | 0.03 | 9 | 2.26 | 0.11 | 0.90 |
|  | 10 | 4.93 | + | + | + | NA | NA | 0.03 | 0.03 | 10 | 5.16 | 0.03 | 0.93 |
|  | 14 | 5.24 | + | + | + | NA | + | 0.03 | 0.04 | 11 | 5.50 | 0.02 | 0.95 |
|  | 1 | 4.70 | + | NA | NA | NA | NA | 0.01 | 0.01 | 6 | 6.77 | 0.01 | 0.96 |
|  | 12 | 4.88 | + | + | + | + | NA | 0.03 | 0.03 | 11 | 7.05 | 0.01 | 0.97 |
|  | 5 | 5.00 | + | NA | NA | NA | + | 0.02 | 0.02 | 7 | 7.13 | 0.01 | 0.98 |
|  | 16 | 5.19 | + | + | + | + | + | 0.03 | 0.04 | 12 | 7.41 | 0.01 | 0.99 |
|  | 3 | 4.67 | + | NA | NA | + | NA | 0.01 | 0.01 | 7 | 8.79 | 0.00 | 0.99 |
|  | 7 | 4.98 | + | NA | NA | + | + | 0.02 | 0.02 | 8 | 9.17 | 0.00 | 1.00 |
|  | 2 | 4.68 | + | NA | + | NA | NA | 0.02 | 0.02 | 9 | 10.94 | 0.00 | 1.00 |
|  | 6 | 4.99 | + | NA | + | NA | + | 0.02 | 0.02 | 10 | 11.40 | 0.00 | 1.00 |
|  | 4 | 4.64 | + | NA | + | + | NA | 0.02 | 0.02 | 10 | 12.90 | 0.00 | 1.00 |
|  | 8 | 4.94 | + | NA | + | + | + | 0.02 | 0.02 | 11 | 13.38 | 0.00 | 1.00 |
| 25(OH)D3 | 12 | 23.18 | + | + | + | + | NA | 0.04 | 0.04 | 11 | 0.00 | 0.18 | 0.18 |
|  | 4 | 23.72 | + | NA | + | + | NA | 0.04 | 0.04 | 10 | 0.43 | 0.14 | 0.32 |
|  | 3 | 23.72 | + | NA | NA | + | NA | 0.02 | 0.02 | 7 | 1.12 | 0.10 | 0.42 |
|  | 10 | 22.29 | + | + | + | NA | NA | 0.03 | 0.03 | 10 | 1.30 | 0.09 | 0.52 |
|  | 2 | 22.83 | + | NA | + | NA | NA | 0.03 | 0.03 | 9 | 1.47 | 0.09 | 0.60 |
|  | 11 | 23.27 | + | + | NA | + | NA | 0.03 | 0.03 | 8 | 1.62 | 0.08 | 0.68 |
|  | 16 | 22.87 | + | + | + | + | + | 0.04 | 0.04 | 12 | 2.00 | 0.07 | 0.75 |
|  | 8 | 23.42 | + | NA | + | + | + | 0.04 | 0.04 | 11 | 2.43 | 0.05 | 0.80 |
|  | 7 | 23.50 | + | NA | NA | + | + | 0.02 | 0.02 | 8 | 3.13 | 0.04 | 0.84 |
|  | 14 | 21.93 | + | + | + | NA | + | 0.03 | 0.03 | 11 | 3.26 | 0.03 | 0.87 |
|  | 6 | 22.48 | + | NA | + | NA | + | 0.03 | 0.03 | 10 | 3.42 | 0.03 | 0.91 |
|  | 15 | 23.06 | + | + | NA | + | + | 0.03 | 0.03 | 9 | 3.65 | 0.03 | 0.94 |
|  | 1 | 22.70 | + | NA | NA | NA | NA | 0.01 | 0.01 | 6 | 3.69 | 0.03 | 0.96 |
|  | 9 | 22.27 | + | + | NA | NA | NA | 0.02 | 0.02 | 7 | 4.49 | 0.02 | 0.98 |
|  | 5 | 22.38 | + | NA | NA | NA | + | 0.01 | 0.01 | 7 | 5.64 | 0.01 | 0.99 |
|  | 13 | 21.97 | + | + | NA | NA | + | 0.02 | 0.02 | 8 | 6.46 | 0.01 | 1.00 |
| 25(OH)D | 4 | 28.37 | + | NA | + | + | NA | 0.03 | 0.03 | 10 | 0.00 | 0.16 | 0.16 |
|  | 2 | 27.52 | + | NA | + | NA | NA | 0.02 | 0.02 | 9 | 0.12 | 0.15 | 0.31 |
|  | 3 | 28.40 | + | NA | NA | + | NA | 0.01 | 0.01 | 7 | 0.37 | 0.13 | 0.44 |
|  | 12 | 28.05 | + | + | + | + | NA | 0.03 | 0.03 | 11 | 1.42 | 0.08 | 0.52 |
|  | 10 | 27.20 | + | + | + | NA | NA | 0.02 | 0.02 | 10 | 1.64 | 0.07 | 0.59 |
|  | 1 | 27.39 | + | NA | NA | NA | NA | 0.01 | 0.01 | 6 | 1.77 | 0.07 | 0.66 |
|  | 8 | 28.43 | + | NA | + | + | + | 0.03 | 0.03 | 11 | 2.09 | 0.06 | 0.72 |
|  | 11 | 28.20 | + | + | NA | + | NA | 0.01 | 0.01 | 8 | 2.20 | 0.05 | 0.77 |
|  | 6 | 27.53 | + | NA | + | NA | + | 0.02 | 0.02 | 10 | 2.20 | 0.05 | 0.82 |
|  | 7 | 28.54 | + | NA | NA | + | + | 0.01 | 0.01 | 8 | 2.42 | 0.05 | 0.87 |
|  | 16 | 28.10 | + | + | + | + | + | 0.03 | 0.03 | 12 | 3.52 | 0.03 | 0.90 |
|  | 9 | 27.23 | + | + | NA | NA | NA | 0.01 | 0.01 | 7 | 3.68 | 0.03 | 0.92 |
|  | 14 | 27.21 | + | + | + | NA | + | 0.02 | 0.02 | 11 | 3.73 | 0.02 | 0.95 |
|  | 5 | 27.45 | + | NA | NA | NA | + | 0.01 | 0.01 | 7 | 3.82 | 0.02 | 0.97 |
|  | 15 | 28.34 | + | + | NA | + | + | 0.01 | 0.01 | 9 | 4.25 | 0.02 | 0.99 |
|  | 13 | 27.29 | + | + | NA | NA | + | 0.01 | 0.01 | 8 | 5.74 | 0.01 | 1.00 |
